# A Meiosis-Specific Form of the APC/C Promotes the Oocyte-to-Embryo Transition by Decreasing Levels of the Polo Kinase Inhibitor Matrimony

**DOI:** 10.1371/journal.pbio.1001648

**Published:** 2013-09-03

**Authors:** Zachary J. Whitfield, Jennifer Chisholm, R. Scott Hawley, Terry L. Orr-Weaver

**Affiliations:** 1 Whitehead Institute, Department of Biology, Massachusetts Institute of Technology, Cambridge, Massachusetts, United States of America; 2 Stowers Institute for Medical Research, Kansas City, Missouri, United States of America; 3 Department of Molecular and Integrative Physiology, University of Kansas Medical Center, Kansas City, Kansas, United States of America; Dana-Farber Cancer Institute, United States of America

## Abstract

Oocytes are stockpiled with proteins and mRNA that are required to drive the initial mitotic divisions of embryogenesis. But are there proteins specific to meiosis whose levels must be decreased to begin embryogenesis properly? The Drosophila protein Cortex (Cort) is a female, meiosis-specific activator of the Anaphase Promoting Complex/Cyclosome (APC/C), an E3 ubiquitin ligase. We performed immunoprecipitation of Cortex followed by mass spectrometry, and identified the Polo kinase inhibitor Matrimony (Mtrm) as a potential interactor with Cort. *In vitro* binding assays showed Mtrm and Cort can bind directly. We found Mtrm protein levels to be reduced dramatically during the oocyte-to-embryo transition, and this downregulation did not take place in *cort* mutant eggs, consistent with Mtrm being a substrate of APC^Cort^. We showed that Mtrm is subject to APC^Cort^-mediated proteasomal degradation and have identified a putative APC/C recognition motif in Mtrm that when mutated partially stabilized the protein in the embryo. Furthermore, overexpression of Mtrm in the early embryo caused aberrant nuclear divisions and developmental defects, and these were enhanced by decreasing levels of active Polo. These data indicate APC^Cort^ ubiquitylates Mtrm at the oocyte-to-embryo transition, thus preventing excessive inhibition of Polo kinase activity due to Mtrm's presence.

## Abstract 2

During the oocyte-to-embryo transition in Drosophila, degradation of the Polo kinase inhibitor, Matrimony, depends on Cortex, a meiosis-specific form of the Anaphase Promoting Complex/Cyclosome that is required for the oocyte's normal transition from meiosis to mitosis.

## Author Summary

Despite their many differences, the meiotic and mitotic divisions of the early embryo take place within the same cytoplasmic space. The oocyte-to-embryo transition is the process by which an oocyte, which initially undergoes meiosis, becomes “adapted” to support the rapid mitotic divisions of embryogenesis. This involves fertilization as well as the stockpiling of proteins and mRNA for the transcriptionally silent early embryo. The Anaphase Promoting Complex/Cyclosome (APC/C) is a large protein complex that is active during both mitosis and meiosis and is responsible for targeting certain proteins for degradation. The discovery of the existence of APC/C activators that are present only during meiosis hinted at the possibility that this complex also functions to regulate protein degradation during the oocyte-to-embryo transition. Here we study Cortex, a female- and meiosis-specific activator of the APC/C in the fruit fly *Drosophila melanogaster*. We find that Cortex activity is necessary for the degradation of Matrimony, a key regulator of female meiosis in Drosophila. Matrimony itself inhibits Polo kinase, another important regulator of both mitosis and meiosis that also functions in chromosome segregation, centrosome dynamics, and cytokinesis. When excess Matrimony protein is not removed from the early embryo, developmental defects arise. Together our findings demonstrate that the precise regulation of Matrimony levels in the egg is necessary for the switch from meiosis to mitosis.

## Introduction

The oocyte-to-embryo transition is the developmental course by which an oocyte not only switches from a meiotic to a mitotic program, but becomes fully competent to support early embryogenesis. Initially, fertilization introduces the haploid genomic content of the sperm into the egg. Egg activation, triggered by fertilization in vertebrates and independent of fertilization in insects, signals the resumption and completion of meiosis in the egg [1],[2]. Following successful completion of meiosis, pronuclear fusion creates a single diploid nucleus from the individual haploid sperm and egg nuclei. The single diploid nucleus must then transition to a mitotic cell cycle within the same cytoplasm in which the meiotic divisions took place.

The oocyte-to-embryo transition can proceed normally only if the preceding events of meiosis are completed successfully. During *Drosophila melanogaster* oogenesis, an oocyte enters prophase I following completion of premeiotic S-phase. After homologous chromosome pairs synapse and recombine, the oocyte enters a prolonged prophase I arrest. Oocyte maturation then releases this primary arrest, allowing the oocyte to continue meiosis until its secondary arrest at metaphase I, in what is known as a stage 14 oocyte. Lastly, egg activation triggers resumption and completion of meiosis concordantly with the oocyte-to-embryo transition itself [1],[2].

The switch from meiosis to mitosis is controlled by cellular proteins and structures produced during gametogenesis, with both the sperm and egg making unique contributions. The centrosome, important for proper spindle formation during mitotic divisions, is brought into the acentrosomal egg by the sperm [3]. The initial rapid divisions of a developing embryo are driven by the maternal stockpile of nutrients, mRNA, and translational machinery that are “packed” into the egg during oocyte differentiation [1]. Additionally, the egg also contains numerous meiosis-specific proteins. These meiosis-specific proteins are crucial for proper meiotic progression, but are not necessarily needed after the switch to mitosis.

There are known examples of proteins uniquely employed in meiosis that need to be removed prior to mitosis [4]. In *C. elegans*, the MBK-2 kinase promotes the oocyte-to-embryo transition. One target is the katanin subunit MEI-1 [5], and phosphorylation of MEI-1 by MBK-2 marks it for degradation before the completion of meiosis [6]. A gain-of-function MEI-1 protein that persists into embryogenesis often leads to a short, mispositioned mitotic spindle [7]. The *Saccharomyces cerevisiae* meiosis-specific protein Spo13 prevents the biorientation of sister chromatids at meiosis I, ensuring homologs segregate together [8],[9]. Spo13 is actively targeted for degradation during anaphase I by the Cdc20 form of the Anaphase Promoting Complex/Cyclosome (APC/C) [10]. Interestingly, a nondegradable form of Spo13 does not result in a significant meiotic phenotype; however, overexpression of Spo13 leads to mitotic cell cycle defects [10],[11],[12]. This demonstrates the necessity of degrading a meiosis-specific protein not for proper meiotic progression, but subsequent mitotic progression.

The unique mechanisms of meiosis such as segregation of homologs in meiosis I, absence of DNA replication between divisions, and the meiotic arrests during oogenesis require either unique regulators or altered control of factors that also are used in mitosis. For example, during mitosis the mitotic cyclins are completely degraded as the cell progresses through the metaphase to anaphase transition and exits from mitosis. In contrast, the mitotic cyclins are left at an intermediate level after the metaphase to anaphase transition of meiosis I; low enough to exit from meiosis I, but high enough to prevent re-replication [13],[14]. This altered control of mitotic regulators may need to be removed upon the start of embryogenesis. The APC/C inhibitor Emi2 is responsible for maintaining Cyclin B1 levels after meiosis I in mouse oocytes, but it is quickly degraded to allow for meiotic exit (though it has been shown to reestablish its levels in early embryogenesis in Xenopus) [15],[16],[17],[18]. This illustrates how normal mitotic cell cycle regulation can be altered through the use of unique meiotic proteins.

Regulated degradation of proteins, particularly by the APC/C, plays an indispensable role in progression through the mitotic and meiotic divisions [19],[20]. The APC/C ubiquitylates numerous proteins during mitosis, targeting them for degradation and promoting mitotic progression and exit. Similarly, during oogenesis proper cell cycle regulation by the APC/C is crucial in maintaining coordination between meiosis and development. The APC/C must use activator proteins (Cdc20/Fizzy and Cdh1/Fizzy-related in mitosis) to recognize its substrates. Interestingly, meiosis-specific activators of the APC/C are known to exist in both budding [21] and fission yeast [22] in addition to sex and meiosis-specific APC/C activators in Drosophila [20],[23],[24]. Elucidating the function and targets of these meiosis-specific APC/C activators will give valuable insights into meiotic regulation and the transition from meiosis into mitosis.

The Drosophila protein Cort is a female, meiosis-specific activator of the APC/C [23],[24],[25]. It is expressed exclusively during oogenesis and is itself targeted for degradation by the APC/C soon after meiotic completion [23]. Cort is dispensable for viability, but absolutely essential for fertility. Eggs laid by *cort* mutant mothers arrest in metaphase II [26]. During Drosophila female meiosis, Cort and Fzy/Cdc20 both contribute to meiotic progression, whereas Fizzy-related/Cdh1 is not believed to play a role. Cort coordinates with Fizzy/Cdc20 during meiosis to degrade the Cyclins [23],[25], but whether it also has other substrates is unknown. Identifying additional substrates of APC^Cort^ will give further insight into the differential regulation of meiosis and mitosis, as well as the necessary steps to transition from oocyte to embryo.

Here we show that degradation of the female-specific protein Mtrm during meiotic completion is dependent on the activity of Cort. Furthermore, we show that this downregulation of Mtrm is crucial to the proper onset of embryogenesis.

## Results

### Cort Binds to the Polo Inhibitor Mtrm

To recover substrates and regulators of APC^Cort^, a functional myc-tagged Cort [23] was immunoprecipitated from whole ovaries, and co-immunoprecipitated proteins were identified by mass spectrometry. In addition to isolating multiple components of the APC/C as expected [23], the Polo inhibitor Mtrm was recovered as a potential substrate/interactor (Table S1). Mtrm was identified initially in a genetic screen for dominant effects on achiasmate chromosome segregation in Drosophila oocytes [27], and it was later shown to function as a direct inhibitor of Polo kinase during meiosis I [28]. Given Mtrm's essential role during female meiosis, we sought to explore further its relationship to Cort.

**Figure 1:**
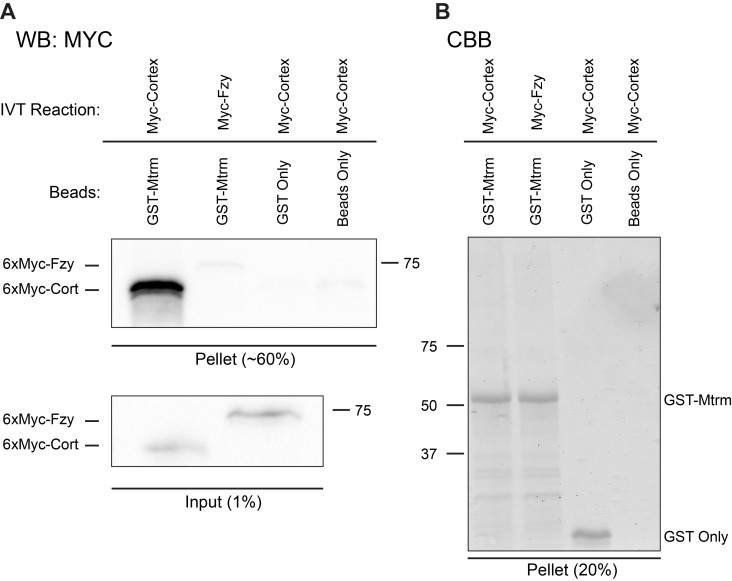
Cort physically interacts with Mtrm *in vitro*. (A) Western blot showing in vitro translated Myc-tagged Cort stably binds to GST-Mtrm, but not to GST only or beads only. In vitro translated Myc-tagged Fzy/Cdc20 is unable to bind GST-Mtrm. About 60% of each pellet sample was subjected to SDS-PAGE followed by Western blotting (remaining pellet sample was used for B). Lower panel shows 1% of total input of *in vitro* translated 6×MycCort and 6×MycFzy/Cdc20. Panels were probed with anti-Myc (9E10) antibody. Molecular weight markers are indicated to the side of the blot. (B) Coomassie stain of purified proteins used in binding assay. 25% of the final washed pellet was subjected to SDS-PAGE followed by Coomassie staining. Molecular weight markers are indicated to the side of the gel.

To confirm the physical interaction between Cort and Mtrm, *in vitro* binding assays were performed. GST-tagged Mtrm and GST alone were expressed and purified from bacteria (Figure 1B), and then incubated with *in vitro* translated 6×Myc-Cort produced in rabbit reticulocyte lysate. Cort strongly bound to GST-Mtrm beads, but not to GST-only beads or beads alone, consistent with the physical interaction between these two proteins being direct (Figure 1A). Moreover, *in vitro* translated Cortex lacking its C-terminus binds GST-Mtrm much less efficiently (Figure S1). The C-terminus of Cortex is made up mainly of its WD40 repeats [24], which are known to mediate substrate binding in other APC/C activators [29]. These data are consistent with Cortex binding Matrimony directly through its WD40 propeller.

Cort and Fzy/Cdc20 are both required for degradation of the mitotic cyclins during female meiosis [23],[25], and therefore share at least a subset of their substrates. We also tested whether the interaction between Cort and Mtrm was specific, or whether Mtrm might be a target of all forms of the APC/C (or an APC/C regulator). In contrast to 6×MycCort, little to no *in vitro* translated 6×MycFzy/Cdc20 bound to GST-Mtrm (Figure 1A). Importantly, *in vitro* translated Fzy/Cdc20 could bind Cyclin A, a known substrate/interactor [30],[31]. Full-length Cortex also bound Cyclin A, albeit to a lesser extent than it binds Matrimony (Figure S1). Thus, the interaction between Cort and Mtrm is specific, suggesting regulation between these two female, meiosis-specific proteins.

### Decreased Mtrm Protein Levels After Meiosis Are Cort Dependent

**Figure 2:**
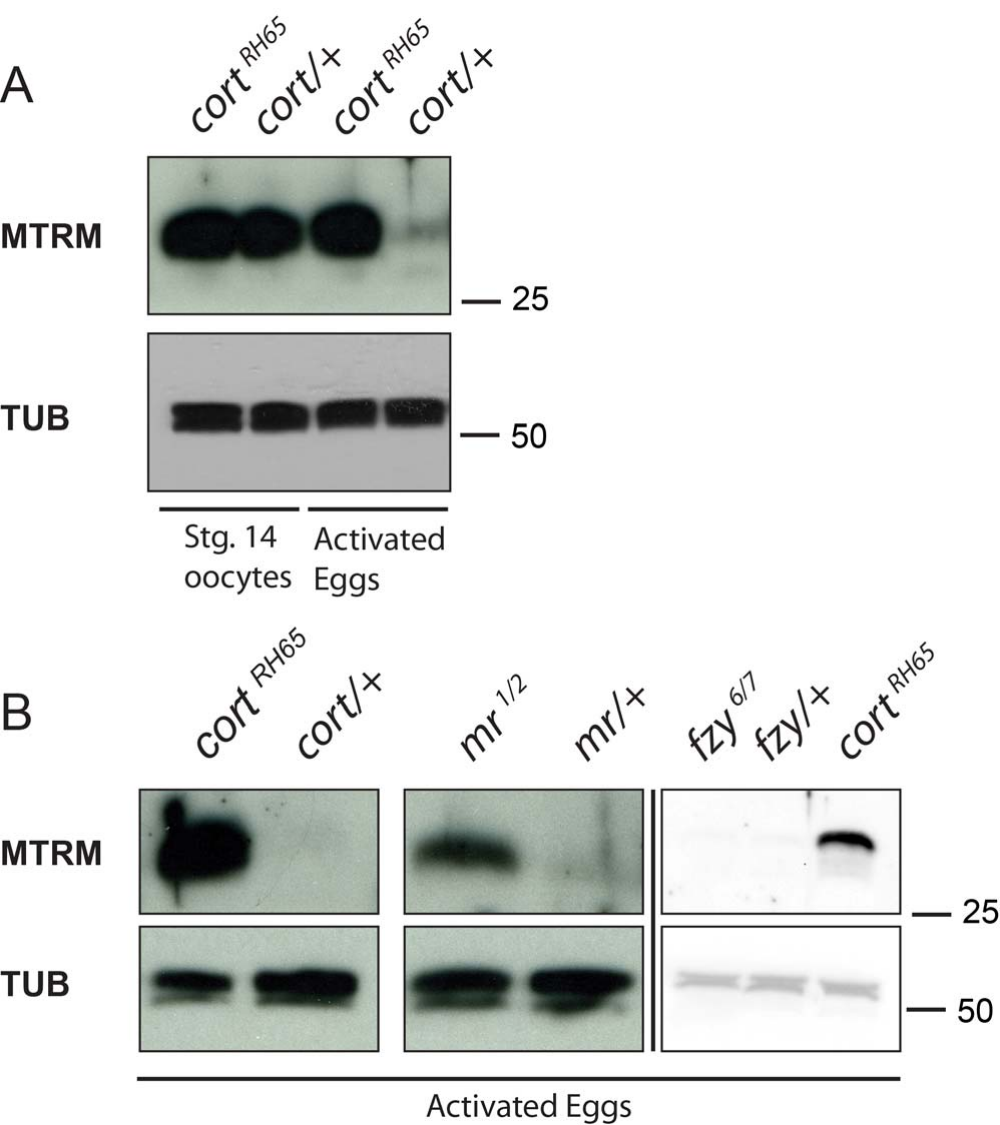
Cort activity is required for Mtrm destabilization.

Mtrm protein levels increase throughout meiosis I [32]. Interestingly, its levels are drastically reduced by the time meiosis is completed (Figure 2A; compare *cort/+* stg. 14 oocyte to *cort/+* activated egg). This pattern of expression mimics that of Cort, which itself is a substrate of the APC/C [23]. As with Cort, such a sharp transition in Matrimony protein levels suggests active degradation, potentially through the action of APC^Cort^.

To test whether the decrease in Mtrm is dependent on Cort function, we compared Mtrm protein levels in *cort* mutant eggs to heterozygous control unfertilized eggs. Unfertilized eggs have completed meiosis, but have not initiated embryogenesis, and therefore provide the best control for *cort* mutant eggs. In contrast to heterozygous unfertilized eggs, activated eggs laid by homozygous *cort* females retained high levels of Mtrm protein, consistent with it being a substrate of APC^Cort^ (Figure 2A,B). Moreover, unfertilized eggs laid by females mutant for *morula/APC2*, a component of the APC/C itself, also showed elevated levels of Mtrm. This shows APC/C function is necessary to trigger the decrease in Mtrm protein (Figure 2B). Importantly, *fzy/cdc20* mutant unfertilized eggs did not show elevated Mtrm levels, again illustrating Mtrm is not a general APC/C substrate (Figure 2B). Together these data demonstrate the decrease in Mtrm protein upon meiotic completion (or during meiosis II) is dependent specifically on APC^Cort^ function. We hypothesized the relatively large pool of Mtrm present in the ovary is necessary for proper progression through meiosis, but such high levels may be detrimental in early embryogenesis.

### Requirement for APC Motif in Mtrm for APC^Cort^-Dependent Destabilization

**Figure 3:**
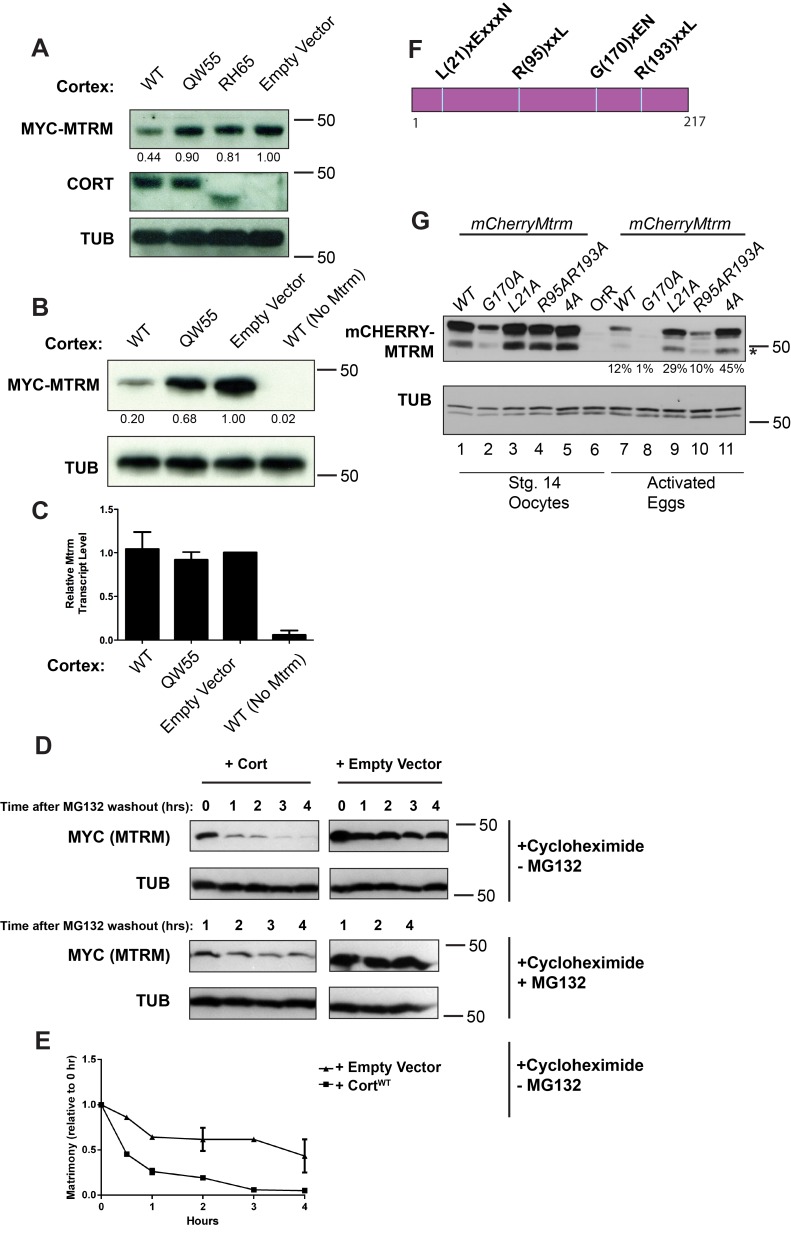
Cort expression leads to proteasome-mediated degradation of Mtrm in cell culture. (A) Western blots showing levels of Mtrm and Cort in transfected Kc167 cells. *pMT-cort* and *pMT-6×myc-mtrm* were transfected into Kc167 cells. The form of transfected Cort is indicated above each lane. Only wild-type Cort leads to decreased levels of tagged Mtrm protein. The *RH65* mutation results in a premature stop codon in Cort. Myc-Mtrm band intensity is quantified below the Myc-Mtrm panel. Band intensity is normalized to tubulin and is expressed relative to empty vector. (B and C) Cells transfected with *pMT-6×myc-mtrm* (lanes 1–3; lane 4 transfected with *pMT-empty* in place of *mtrm*) and the indicated form of Cort (WT, QW55, or *pMT-empty*) were split and subjected to both Western blot (B) and quantitative PCR (C). Myc-Mtrm band intensity is quantified as in (A). For qPCR, *mtrm* transcript levels are normalized to *actin5c* and shown relative to empty vector. (D) Western blot showing Mtrm protein levels over time. Time indicates hours post-MG132 washout. Rate of Matrimony degradation is faster in the presence of Cortex versus empty vector. The rate of degradation is slowed in continued presence of MG132. (E) Quantification of –MG132 blot in (D). The 1-, 2-, and 4-h time points are averages of two experiments. Mtrm amount is normalized to tubulin and shown relative to amount at the 0 h time point. (F) Illustration of candidate APC/C recognition motifs. (G) The L21A mutation stabilizes mCherry-Mtrm in embryos. Western blots of stage 14 oocytes and fertilized eggs (1 h collection) are shown. The 4A mutant consists of L21A, R95A, R193A, and H94A (a mutation in a possible APC/C initiation motif [54]). Percentage below mCherry activated egg lanes indicates remaining protein left, normalized to tubulin, and relative to amount at stage 14. Asterisk denotes cleavage product due to hydrolysis of acylimine linkage in the mCherry tag [55]. Myc-Mtrm was detected using anti-Myc antibody (A, B, D) and mCherry-Mtrm was detected using anti-RFP (G).

We exploited Drosophila cell culture to study the effects of Cort on Mtrm stability, as it permits the expression of proteins in an easily manipulated system. Neither Cort nor Mtrm is expressed endogenously in Drosophila Kc167 cell culture cells, but both can be expressed transiently through transfection (Figure 3A). In a stable cell line expressing Cort, Cyclin A protein levels were decreased markedly and Cyclin B levels marginally (Figure S2A), indicating functional APC^Cort^. The changes in mitotic Cyclin protein levels did not detectably affect cell cycle progression, however, as measured by the mitotic index (Table S2) and FACS analysis (Table S3).

If Mtrm is targeted for degradation by APC^Cort^, levels of Mtrm protein should be reduced in the presence of Cort. Indeed, levels of a Myc-tagged Mtrm were reduced when functional Cort was expressed (Figure 3A). Moreover, expression of functionally null alleles of Cort, Cort^QW55^ (a missense mutation) or Cort^RH65^ (a nonsense mutation) [24],[26],[33], failed to decrease Mtrm protein levels. Therefore, wild-type Cort function is required to bring about the observed decrease in Mtrm protein. Consistent with APC^Cort^ affecting Mtrm levels through degradation, these cells contained similar amounts of *mtrm* transcript, illustrating the effect is posttranscriptional (Figure 3B and C). Additionally, reduction of Mtrm levels was not observed when a 6×Myc-tagged Fzy/Cdc20 was expressed, again showing the selectivity of APC^Cort^ for Mtrm (Figure S2B).

We next used this cell-culture-based system to determine whether APC^Cort^'s effect on Mtrm was truly the result of degradation. Mtrm protein was accumulated during arrest with the proteasome inhibitor MG132, and upon release of the arrest translation was inhibited with cycloheximide and Mtrm protein levels examined over time in the presence or absence of Cortex (Figure 3D,E). Mtrm protein levels decreased rapidly in the presence of Cort. Importantly, this continued decrease was abolished in the continued presence of MG132 to inactivate the proteasome. Mtrm levels remained higher when an empty vector was transfected in place of Cort. These data establish that APC^Cort^ affects Mtrm levels through proteasome-mediated degradation.

Given the decrease in Mtrm is mediated through degradation, we searched Mtrm's primary amino acid sequence for APC/C recognition motifs that could influence its stability during the oocyte-to-embryo transition. Four motifs previously implicated in APC/C-mediated degradation [19] are present within Mtrm's 217 amino acid sequence (Figure 3F). To examine the role these motifs play in Mtrm protein stability at the oocyte-to-embryo transition, transgenic flies expressing mCherry-Mtrm under the control of *mtrm*'s endogenous promoter were created. mCherry-Mtrm protein levels decreased at the oocyte-to-embryo transition as expected (Figure 3G, lanes 1 and 7). Point mutants in the four candidate APC motifs were also examined for their effect on (mCherry-) Mtrm protein stability. Whereas the G170A mutation and the double R95A/R193A mutations did not stabilize mCherry-Mtrm in activated eggs (Figure 3G, compare lanes 2 and 8; 4 and 10, respectively), mutation of leucine 21 exhibited partial stabilization (Figure 3G, lanes 3 and 9). A quadruple mutant of mCherry-Mtrm that also contains the L21A mutation is partially stabilized as well (Figure 3G, lanes 5 and 11). Importantly, both Mtrm-L21A and Mtrm-4A are functional, as judged by their ability to rescue *mtrm^+/^* ^−^ induced nondisjunction (Table S4). Given mCherry-Mtrm-L21A is still partially degraded at the oocyte-to-embryo transition, L21 is not likely to be the only residue responsible for Matrimony degradation. It is intriguing to note, however, that L21 is part of the LxExxxN APC/C destruction motif found within Spo13, another meiosis-specific substrate of the APC/C [10].

### Genetic Interactions Reveal an Antagonistic Relationship Between Cort and Mtrm

We next investigated the genetic relationship between Cort and Mtrm, specifically in the background of a mutant with low APC^Cort^ activity. Mutants with low APC^Cort^ activity arrest without completing meiosis, presumably due to a failure to degrade key substrates. If Mtrm were such a substrate, we hypothesized that decreasing its levels could lead to suppression of the reduced APC^Cort^ phenotype. All alleles of *cort* are null [26], however mutation of Cort's dedicated transcription factor *grauzone* results in decreased levels of *cort* transcript [34] and protein [23]. Activated eggs laid by *grauzone* mutant females also arrest in meiosis II (just as *cort* eggs do) [26], thus illustrating that such low levels of APC^Cort^ cannot efficiently cause degradation of key substrates. Decreasing levels of the Mtrm substrate may be sufficient to permit progression past the meiotic arrest. Alternatively, the reduced levels of one key substrate may afford low APC^Cort^ enough opportunity to target its remaining substrates for degradation. Thus we used this sensitized background to test whether decreased *mtrm* permitted progression past the *grauzone* metaphase II arrest.

**Figure 4:**
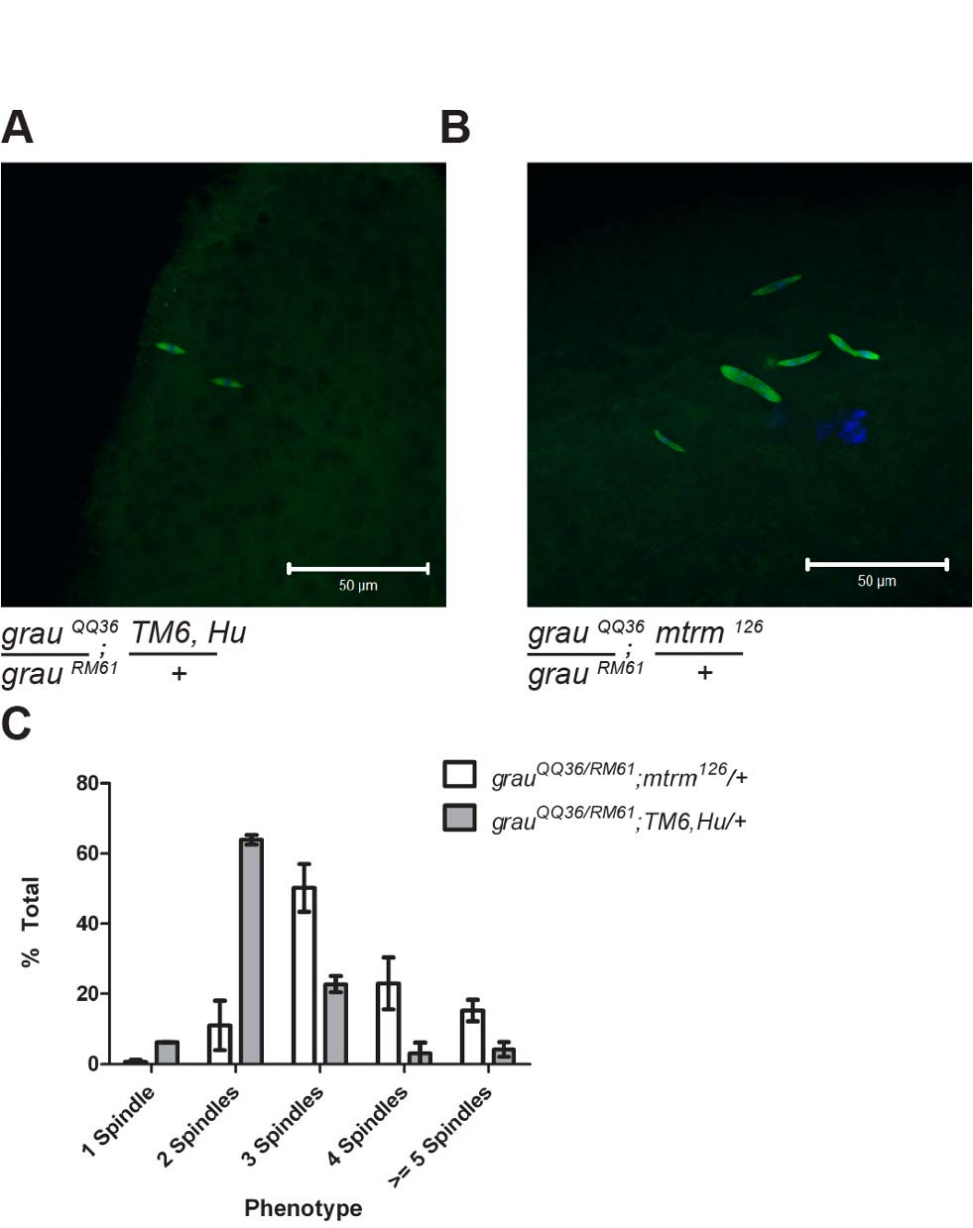
*cort* and *mtrm* show an antagonistic relationship in vivo. (A and B) Fertilized eggs from females of the indicated genotypes are shown. When *mtrm* is mutated in conjunction with *grauzone*, an increased number of spindles is observed. Even mutation of a single copy of the *mtrm* gene dominantly suppresses the *grauzone* phenotype. Tubulin is shown in green and DNA in blue. Scale bar indicates 50 um. (C) Quantification of eggs from (A) and (B). The *TM6* balancer siblings served as the wild-type control for *mtrm*. *n* = 167 for *grau^QQ36^/^RM61^;mtrm^126^/+* and *n* = 67 for *grau^QQ36^/^RM61^;TM6/+*.

Strikingly, when one copy of the *mtrm* gene was removed, we observed partial suppression of the *grau* phenotype. *grau* eggs typically arrest with two spindles at metaphase II, but *grau* eggs also mutant for one copy of *mtrm* contained, on average, an increased number of spindles (Figure 4). These spindles appear acentriolar, and thus are likely not mitotic. Supporting this, no gamma-tubulin (a common component of centrosomes) is present at the spindle poles (Figure S3). These spindles likely arise from completion of meiosis followed by all meiotic products (including polar bodies) forming bipolar spindles and possibly dividing, reminiscent of the effect of *polo* mutation on meiosis II [35]. Importantly, the observed increase in spindle number is not due to a restoration of Cort protein levels (Figure S4). Thus the *mtrm* mutation partially suppresses the *grau* phenotype, allowing further progression through the oocyte-to-embryo transition.

### Increased Mtrm Levels in the Embryo Lead to Developmental Defects

Proteins and mRNA deposited into the oocyte during oogenesis control the early embryonic divisions, but it is possible some of these proteins function in meiosis and then need to be removed. We hypothesized degradation of Mtrm at the oocyte-to-embryo transition by APC^Cort^ is a crucial step necessary to ensure proper development of the syncytial embryo. To test this hypothesis, we overexpressed a transgenic *mtrm* using the *UAS-GAL4* system. 3×FLAG-Mtrm was overexpressed in the ovary using the maternal alpha tubulin driver, resulting in excess Mtrm being present in the early embryo (Figure S5A/B).

**Figure 5:**
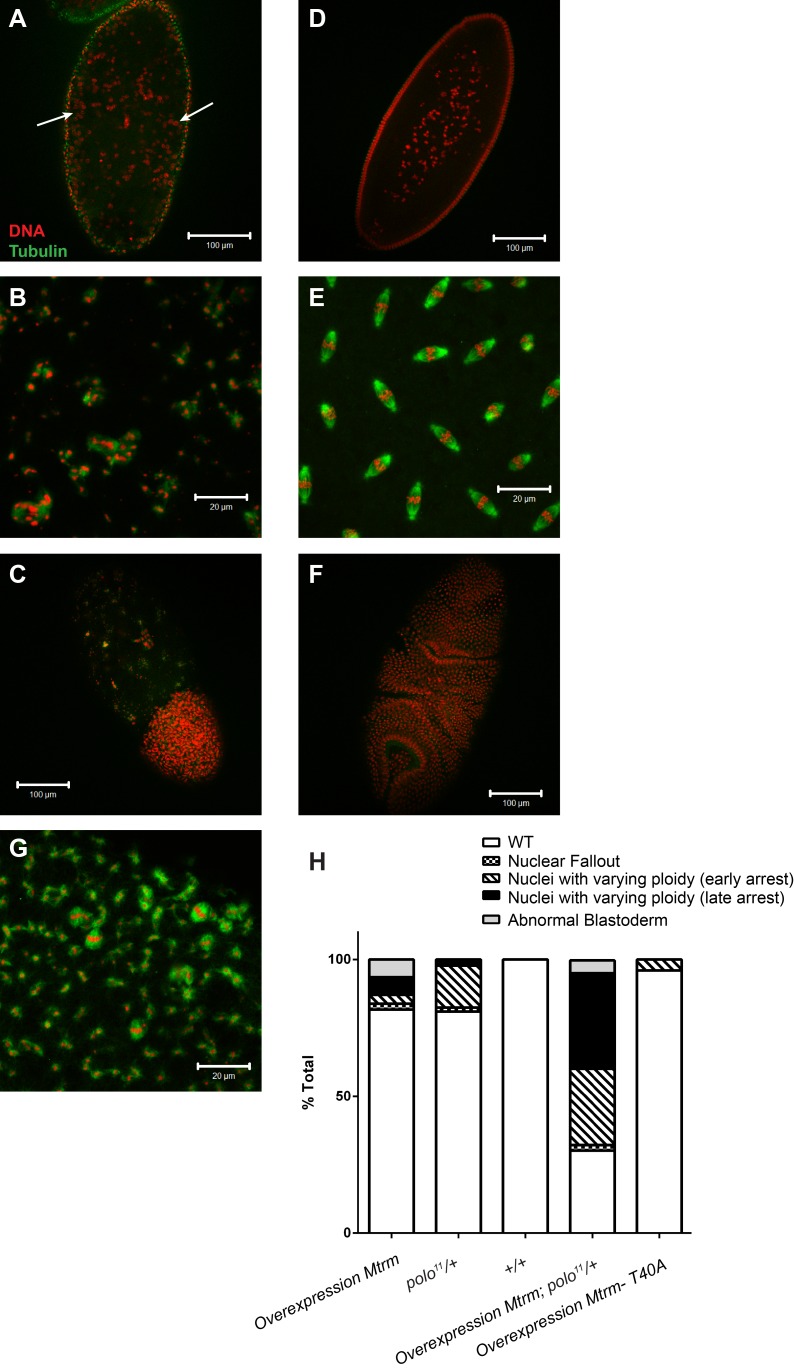
Developmental defects result from increased Mtrm expression. (A–C) Representative images of fertilized eggs laid by females overexpressing 3×FLAG-Mtrm using the *MATalpha4-GAL-VP16* driver. (A) Embryo undergoing “nuclear fallout.” Nuclei can be seen having fallen below the surface of the embryo (white arrows). (B) An embryo showing scattered DNA with disorganized tubulin. (C) An embryo that underwent uneven development across its length, showing abnormal development up to the blastoderm stage. (D–F) Control fertilized eggs showing proper development at comparable stages to those in (A–C). Scale bar indicates 100 um in (A), (C), (D), and (F). It indicates 20 um in (B), (E), and (G). (G) A fertilized egg laid by females overexpressing 3×FLAG-Mtrm and heterozygous for *polo^11^*. These embryos predominantly had scattered DNA and disorganized tubulin. (H) Quantification of embryos shown in (A–G). The genotype for Overexpression Mtrm is *UAS3×FLAGmtrm/+; P{matα4-GAL-VP16}V37/+* (*n* = 93), the genotype for *polo^11^/+* is *polo^11^/P{matα4-GAL-VP16}V37* (*n* = 137), +/+ is the control for driver alone and is *TM6,Sb/P{matα4-GAL-VP16}V37* (*n* = 109), the genotype for Overexpression Mtrm;*polo^11^/+* is *UAS3×FLAGmtrm/+; polo^11^/P{matα4-GAL-VP16}V37* (*n* = 86), and the genotype for Overexpression Mtrm-T40A is *UAS3×FLAGmtrm-T40A/+; P{matα4-GAL-VP16}V37/+* (*n* = 45).

This surplus of Mtrm caused a variety of defects in early embryogenesis, which we categorized into three phenotypes (Figure 5A–C). We observed some embryos undergoing nuclear fallout (Figure 5A). During nuclear fallout, nuclei at the surface of an embryo that have detached from their centrosomes fall back into the middle of the embryo [36]. We also found embryos that exhibited complete mitotic catastrophe (Figure 5B), showing only scattered DNA with no real spindle organization. DNA masses seemed to contain varying chromosomal content, and were usually associated with tubulin. These embryos were found with variable amounts of total DNA, some containing DNA over the entire expanse of the embryo (late arrest), while others only contained DNA in a particular section of the embryo (early arrest). Lastly, some embryos showed scattered DNA/tubulin over a portion of the embryo, whereas the rest of the embryo appeared to reach the blastoderm stage (Figure 5C). These embryos seemingly underwent an abortive/abnormal development up to the blastoderm stage. Given the centrosome's crucial role in spindle organization and the requirement for Polo kinase for proper centrosome attachment in the early embryo [37], there are many ways these phenotypes could be obtained. In summary, these data illustrate that the downregulation of Mtrm protein following meiosis is biologically significant to early embryonic development.

The defects observed from *mtrm* overexpression likely result from low Polo kinase activity, given Mtrm's known function as its inhibitor. If true, mutating *polo* should further exacerbate the *mtrm* overexpression phenotype. Indeed, overexpression of Mtrm in conjunction with heterozygous *polo^11^* results in a substantially higher proportion of defective embryos (Figure 5H). Additionally, the observed defects are often more severe, with DNA completely fragmented and tubulin in almost random configurations (Figure 5G). In our hands the heterozygous *polo^11^* mutation alone also exhibited defects similar to *mtrm* overexpression alone, but these fell primarily into one phenotypic category (Figure 5H). These data are consistent with increased Mtrm in the early embryo causing developmental defects due to excessive inhibition of Polo kinase activity (and potentially other, unknown targets).

To address the possibility that Matrimony affects proteins other than Polo, we expressed a mutant form of Matrimony deficient in Polo binding. Mtrm-T40A is unable to bind Polo, and cannot rescue chromosome nondisjunction in *mtrm/+* heterozygotes [28],[38]. In contrast to wild-type Matrimony, expression of Mtrm-T40A did not cause any developmental defects (Figure 5H). Importantly, expression of both the WT and T40A transgenes is similar using the maternal alpha tubulin driver (Figure S5C). Thus, high levels of Matrimony in the early embryo cause developmental defects due to inhibition of Polo kinase activity.

## Discussion

Despite its pivotal role in development, regulation of the oocyte-to-embryo transition is poorly understood. Given the maternal stockpiles in the oocyte, mechanistic differences between meiosis and mitosis, and meiosis-specific forms of the APC/C, it is crucial to determine which proteins need to be degraded to switch correctly from meiosis to mitosis. The meiosis-specific activator Cort is essential for the transition from oocyte to embryo despite Fzy/Cdc20's presence. Cortex's existence raised the possibility that degradation of particular meiosis-specific proteins may be necessary for the onset of embryogenesis. Here we show this to be the case: the Cort form of the APC/C is required for Mtrm's destruction at the oocyte-to-embryo transition. Furthermore, reduced levels of Mtrm heading into embryogenesis are necessary for proper development, indicative of requirements for differential levels of the protein in meiosis and mitosis.

A requirement for reduction in levels of Mtrm is illustrated by the deleterious effects of overexpression of the protein in the embryo. A crucial role for Mtrm degradation in the transition from oocyte to embryo is supported by the observation that reduction in levels of Mtrm protein can suppress the developmental block caused by low activity of Cort. In the *grau* mutants, levels of Cort are reduced, and the mutant oocytes arrest in meiosis. By mutating a single copy of the *mtrm* gene, this arrest was overcome, the eggs progressed, and several nuclear divisions occurred.

Mtrm provides key insights into how protein degradation can be regulated at the oocyte-to-embryo transition. Mtrm is not completely removed from the embryo, illustrating that its protein levels are important and degradation does not have to be an all-or-none process. In this case, APC^Cort^ acts as a rheostat, allowing for high levels of Mtrm in meiosis and low levels in mitosis. Consistent with this, it is interesting that stabilized forms of Mtrm (Figure 3G) present at lower levels than the overexpressed wild-type form (Figure S5A/B) did not exhibit an embryonic phenotype (unpublished data). mCherry-Mtrm also is present at levels lower than endogenous Mtrm in stage 14 oocytes, and therefore may never reach high enough levels to be able to cause the developmental defects seen with the overexpressed form of Mtrm. This offers evidence for a specific threshold of Mtrm that can be tolerated in the early embryo.

Polo kinase is a critical regulator of both mitosis and meiosis, and is conserved from yeast to humans. *polo* (and its orthologs) help regulate mitotic/meiotic entry, chromosome segregation, centrosome dynamics, and cytokinesis [39]. With such diverse roles during mitosis and meiosis, Polo function must be carefully regulated. Up-regulation of human Polo-like kinase (Plk1) is prevalent in many human cancers, and identifying potent inhibitors of Plk1 is the focus of much research [40]. In Drosophila, without inhibition by Mtrm during prophase of meiosis I, Polo prematurely triggers nuclear envelope breakdown (through activation of the Cdc25 phosphatase) and eventually leads to chromosome nondisjunction [28]. Mutation of *polo* has direct consequences on female meiotic progression as well. During Drosophila embryogenesis, expression of Scant, a hyperactive form of the Polo antagonist Greatwall kinase, leads to dissociated centrosomes from prophase nuclei [37]. Embryos homozygous for *polo^1^* show a wide array of defects, including irregular DNA masses with disorganized spindles [35], reminiscent of our *mtrm* overexpression phenotype (Figure 5). These data illustrate the importance of Polo kinase in both mitosis and meiosis, and that improper regulation of its activity can have disastrous consequences on cell division.

Current evidence suggests that Mtrm regulates Polo activity during both meiosis and mitosis [28],[32],[37]. Our results shed light on how the oocyte/embryo might use the same protein to regulate Polo during such drastically different cell divisions. Our data indicate meiosis requires high levels of Mtrm protein/Polo inhibition, while low levels of Mtrm are needed for early embryogenesis. This is likely a mechanism to allow for fine tuning of Polo activity during the rapid divisions of the syncytial embryo.

The results here provide an interesting biological counterpoint to a recent study on the *S. cerevisiae* meiosis-specific APC/C activator Ama1. Previously, Ama1 had been known to act later in meiosis, regulating spore formation and Cdc20 degradation at meiosis II [21],[41]. Okaz et al. showed APC^Ama1^ also acts earlier in meiosis to clear out mitotic regulators (including Polo/Cdc5) during the extended meiotic prophase I. Consequently, cells lacking Ama1 exit prematurely from prophase I [42]. It is interesting that two meiosis-specific APC/C activators have now been tied to regulation of Polo kinase. Ama1 has a direct, inhibitory effect early in meiosis, whereas Cort seemingly activates Polo indirectly through degradation of Mtrm late in meiosis.

Mtrm is not likely to be the only specific substrate of Cort, and it will be exciting to search for more APC^Cort^ substrates in the future. It will also be interesting to examine whether Cort targets continue to follow a graded versus all-or-none pattern of degradation during the oocyte-to-embryo transition. Further study of meiosis-specific APC/C activators will give valuable insight into the distinctions between meiotic and mitotic regulation and the control of the onset of embryogenesis.

## Materials and Methods

### Fly Stocks

The *grau^RM61^*, *grau^QQ36^*, *cort^RH65^*, *cort^QW55^* [24],[26],[33], *mtrm^126^* [28], *mr^1^*,*mr^2^* [43],[44], *twine^HB5^* [33],[45], *polo^11^* [37],[46], and *fzy^6^*, *fzy^7^* [47] alleles have all been described. The UASp *myc-cort* transgenic lines were generated previously [23] and were driven by *w-;nanos-GAL4:VP16* [48]. The *UASp-3×FLAG-mtrm^WT^*, *UASp-3×FLAG-mtrm^T40A^*, and *mCherry-mtrm^WT^* (driven by its genomic promoter) were generated previously [28],[38]. *mCherry-mtrm^4A^*, *mCherry-mtrm^L21A^*, *mCherry-mtrm^G170A^*, and *mCherry-mtrm^R95/R193A^* were generated for this study (see below). w^*^; P{matα4-GAL-VP16}V37 was obtained from Bloomington Stock Center (BL 7063). *Oregon R* was used as a wild-type control. Flies were maintained at 22 or 25°C [49].

### Transgenic Lines

To construct the *mtrm^FL^* constructs driven by the genomic *mtrm* promoter, the following fragments were generated by PCR from a wild-type *mtrm* construct and *pFPV-mCherry* (a gift from the Susan Abmayr lab) and ligated into *pBluescriptSKII+*: *BamH*I-*mtrm* 5′UTR-*Avr*II, *Avr*II-*mCherry*-*Pac*I, *Pac*I-*mtrm* + 3′UTR-*Xho*I. The Stowers Molecular Biology facility deleted the *Avr*II and *Pac*I sites using the Stratagene QuikChange II XL Site-Directed Mutagenesis Kit. The Stowers Molecular Biology facility made the point mutations using the Stratagene QuikChange II XL Site-Directed Mutagenesis Kit.

The insert was digested and ligated into *pCasPeR4-attB*, and the sequence verified. The *pCasPeR4-attB-mtrm* constructs were injected into *y,w; attP40* embryos, and integrations into the attP40 site were recovered.

### IP-Mass Spec

Whole ovaries were dissected from 100 to 200 fattened females containing the *UASp-myc-cort* transgene being driven by *nanos-GAL4*. Ovary protein extracts were made by homogenizing in homogenization buffer (25 mM HEPES [pH 7.5], 0.4 M NaCl, 0.1 mM EDTA, 0.1 mM EGTA, 1 mM PMSF, 10% glycerol, complete mini EDTA-free protease inhibitors, 1 tablet/10 ml [Roche]). 110 µl Protein G magnetic bead slurry was coupled (and/or crosslinked using dimethylpimelimidate [Sigma]) to 27.5 µl anti-Myc [9e10] antibody or mouse random IgG. Whole ovary extract was split evenly and incubated with the anti-Myc or random IgG beads for 3 h at 4°C. Beads were then washed in IP buffer (25 mM HEPES [pH 7.5], 100 mM NaCl, 1 mM EGTA, 0.1% Triton X-100, 10% glycerol, complete mini EDTA-free protease inhibitors, 1 tablet/10 ml [Roche]) once, IP buffer + 0.5 M NaCl once, then washed in IP buffer four more times. Bound proteins were eluted in sample buffer. Immunoprecipitated proteins were resolved by SDS-PAGE and silver stained. Bands were cut from the silver stained gel and reduced, alkylated, and digested with trypsin. The resulting peptides were extracted and the volume reduced to 15 µl. The digestion extracts were analyzed by HPLC/tandem mass spectrometry using a Waters NanoAcquity UPLC system and a ThermoFisher LTQ linear ion trap mass spectrometer operated in a data-dependent manner. Tandem mass spectra were extracted by Extract_MSn. Charge state deconvolution and deisotoping were not performed. All MS/MS samples were analyzed using Mascot (Matrix Science, London, UK; version 2.4.0). Mascot was set up to search the refseq_fly_lc2_042413 database (27,878 entries) assuming the digestion enzyme trypsin. Mascot was searched with a fragment ion mass tolerance of 1.00 Da and a parent ion tolerance of 3.0 Da. Iodoacetamide derivative of cysteine was specified in Mascot as a fixed modification. Oxidation of methionine was specified in Mascot as a variable modification. Scaffold (version Scaffold_4.0.5, Proteome Software Inc., Portland, OR) was used to validate MS/MS-based peptide and protein identifications. Peptide identifications were accepted if they could be established at greater than 95.0% probability by the Peptide Prophet algorithm [50]and contained at least two identified peptides. Protein probabilities were assigned by the Protein Prophet algorithm [51]. Proteins that contained similar peptides and could not be differentiated based on MS/MS analysis alone were grouped to satisfy the principles of parsimony.

### Westerns/Immunoblots

Whole ovaries and staged egg chambers were hand dissected from fattened females and homogenized in NP-40 lysis buffer (150 mM NaCl, 50 mM Tris, pH 8.0, 2.5 mM EDTA, 2.5 mM EGTA, 1% NP-40, 1 mM PMSF, complete mini EDTA-free protease inhibitors, 1 tablet/10 ml [Roche]). Unfertilized eggs were obtained by mating virgin females of the indicated genotype to sterile *twine^HB5^* males and collecting for 2 h (or O/N in the case of *mr* females). The eggs were then dechorionated in 50% bleach and homogenized in NP-40 lysis buffer. Protein lysates were spun at 14,000 RPMs for 15 min at 4°C, and supernatant was used as protein sample. Equal protein amount was loaded on 10% SDS-PAGE gels as determined with Bradford reagent (BioRad). Protein was transferred to Immobilon-P membranes (Millipore).

Antibodies used in this study were guinea pig anti-Mtrm (1∶1,000) [28], mouse anti-CycA (1∶50) (Developmental Studies Hybridoma Bank), mouse anti-CycB (1∶50) (Developmental Studies Hybridoma Bank), rat anti-tubulin (yol1/34 and yl1/2) (1∶400–1∶1,000) (Novus Biologicals), guinea pig anti-Cort (1∶2,000) [23], and mouse anti-Myc 9E10 (1∶400–1∶1,000) (Covance). Mouse anti-RFP 3F5 (Chromotek) (1∶500) was used to detect mCherry. Secondary antibodies used were Peroxidase-conjugated anti-mouse, Peroxidase-conjugated anti-guinea pig, and Alkaline Phosphatase-conjugated anti-rat (1∶10,000; Jackson ImmunoResearch).

### *In Vitro* Binding Assays

*In vitro* binding assays using purified GST-Mtrm were done essentially as described [52], with some adjustments. *mtrm* cDNA (LD47919) was cloned into pGEX6p-1 (GE Healthcare) for expression of GST-Mtrm. *6×myc-cort, 6×myc-cort ΔWD40*, and *6×myc-fzy/cdc20* cDNAs were cloned into pOT2. *cortΔWD40* encodes the first 444 nucleotides of the *cort* ORF, followed by a stop codon (TGA). *In vitro* transcription/translation was done using the TnT T7 Coupled Reticulocyte Lysate System (Promega) according to the manufacturer's instructions. 5 µl of the *in vitro* translation reaction was added to beads in 500 µl IP buffer [52] and rotated for 2 h at 4°C. Beads were washed 3× in IP buffer, and bound proteins were eluted with 40 µl 2× sample buffer. 10 µl was analyzed by Coomassie to check levels of GST-tagged proteins, and 25 µl was analyzed by SDS-PAGE/Western blotting.

### Cell Culture, Transfection, qPCR, and Cell Cycle Analysis

Kc167 Drosophila cell culture cells were maintained at 25°C in Schneider's serum media (Invitrogen) supplemented with 10% FBS (Sigma) and 50 ug/ml Pen/Strep.

*pMT-6×myc-mtrm* and *pMT-cort* were generated by cloning the respective constructs into pMT-puro under control of the metallothionein promoter. Kc167 cells were transfected with the indicated constructs using Cellfectin II (Invitrogen) according to the manufacturer's instructions. 48 h after transfection, protein expression was induced with 0.5 mM CuSO_4_ for 1–3 d. After induction, protein lysate was prepared by homogenizing cells in NP-40 lysis buffer for SDS-PAGE/Western as described.

The Cort stable line was generated by transfecting Kc167 cells with pMT-Cort as above, and selecting for stable transfectants with puromycin (5 ug/ml) over multiple passages for ∼3 wk.

For quantitative PCR, transfected Kc cells from a T25 (5 ml) flask were resuspended in 2 ml of 1×PBS. 1.2 ml was used to make protein extract as described and subjected to immunoblotting. 800 µl was used to isolate total RNA for absolute qPCR. Primers against *mtrm* were used to measure transgene expression, and primers against *act5C* were used for normalization. Quantitative PCR was performed using PerfeCTa SYBR Green FastMix (Quanta BioSciences) and analyzed on 7300 qPCR system software (Applied Biosystems).

For cell cycle analysis, the Cortex stable line or Kc cells alone were grown with or without CuSO4 for 1 or 2 d. For proteasome inhibition, MG132 was added to 25 uM 8 h before cells were to be fixed. Kc cells were first washed in 5 ml 1×PBS and resuspended in 500 ul PBS. Cells were then transferred into 4.5 ml ice cold 70% ethanol and rotated for 2 h at 4°C. Fixed cells were kept at −20°C until used for cell cycle analysis. Cells were pelleted at 2000 RPMs for 5 min and washed 2× in 5 ml PBS (once in PBS, spun 2000 RPMs for 10 min). Cells were resuspended in 500 ul PBS containing 50 ug/ml propidium iodide, 0.15% Triton X-100, and 100 ug/ml RNAse A, and rotated O/N at 4°C. Cells were then filtered and run on a FACScan 1 system (BD), and data were analyzed with FlowJo software.

### Mtrm *in Vivo* Degradation Time Course

Kc167 cells in T75 flasks were transfected as above with *pMT6×myc-mtrm* and either *pMT-cort* or *pMT-empty* vector. 48 h after transfection, CuSO_4_ was added to the medium at a final concentration of 0.5 mM. At the same time, MG132 (EMD Chemicals) was added to the media to 25 uM. After 8 h of induction/treatment, cells were washed twice with serum media to remove MG132 and CuSO_4_ and then resuspended in 7 ml serum media. 700 ul of resuspended cells were added to 5 ml fresh media in T25 flasks containing 100 uM cycloheximide (Sigma-Aldrich) with or without MG132 (25 uM). Cells were allowed to grow for the indicated amounts of time, and then were harvested for protein extraction/Western blotting as above.

### Nondisjunction Assays

Nondisjunction assays were carried out as in Bonner et al. [38].

### Embryo Collection and Immunofluorescence

Females were allowed to lay eggs for 2 h (Figure 4) or 1–2 h with 3 h aging (Figure 5). Eggs were prepared for immunofluorescence as described [23]. Kc167 cells were prepared for immunofluorescence essentially as described [53], using concanavalin-A coated slides and 4% formaldehyde as a fixative.

Propidium iodide (Figure 5) or DAPI (Figure 4) was used to stain DNA and anti-alpha tubulin [DM1A]-FITC (1∶250) or anti-alpha tubulin (yol 1/34) 1∶500 was used to visualize microtubules. Anti-gamma-tubulin (GTU-88; Sigma-Aldrich) was used at 1∶500 to visualize gamma-tubulin on centrosomes. Anti-phospho-H3 (Rabbit polyclonal; Upstate/Millipore) was used at 1∶300. When appropriate, secondary antibodies used were Alexa-488 anti-rat and Alexa-568 anti-mouse (1∶1,000; Life Technologies).

### Accession Numbers

The FlyBase accession numbers for genes discussed in this paper are *cortex* (FBgn0000351), *grauzone* (FBgn0001133), *matrimony* (FBgn0010431), *polo* (FBgn0003124), and *fizzy* (FBgn0001086).

## Supporting Information

Figure S1 ***In vitro*** **binding assays with Cyclin A and CortΔWD40.** (A) Western blot showing *in vitro* translated Myc-tagged Fzy/Cdc20 stably binds to GST-CycA. Myc-Cortex also binds, but somewhat less efficiently. Myc-CortexΔWD40 (AA 1–148 of Cortex) is impaired in its ability to bind GST-Mtrm. Glutathione beads alone serve as a negative control. Quantification indicates Myc-Fzy binds to GST-CycA 155× better than to GST-Mtrm. 6×Myc-Cortex (full length) binds GST-Mtrm 5.8× better than 6×Myc-CorttΔWD40. About 60% of each pellet sample was subjected to SDS-PAGE followed by Western blotting (remaining pellet sample was used for B). Right side of panel shows 1% of total input of *in vitro* translated 6×MycCort, 6×MycFzy/Cdc20, and 6×Myc-CortΔWD40. Blot was probed with anti-Myc (9E10) antibody. Molecular weight markers are indicated to the side of the blot. (B) Coomassie stain of purified proteins used in binding assay. 20% of the final washed pellet was subjected to SDS-PAGE followed by Coomassie staining. Molecular weight markers are indicated to the side of the gel.
(TIF)

Figure S2 **Levels of cell cycle proteins in cell culture system.** (A) A cell line with a stable *cort* gene shows decreased Cyclin protein levels. Western blots comparing levels of indicated proteins in a *cort* stable line and cells transfected with *pMT-eGFP* instead. Both populations were also transfected with *pMT-6×myc-mtrm*. Molecular weight markers are indicated to the side of the blot. (B) Expression of myc-tagged Fizzy/Cdc20 does not decrease myc-tagged Mtrm levels. Amount of plasmid used to transfect cells is indicated above each lane. Cells were also transfected with equal amounts of *pMT-6×myc-mtrm* (except last lane). The asterisks indicate nonspecific bands. Both Myc-Fzy and Myc-Mtrm were detected using anti-myc antibodies. Molecular weight markers are indicated to the side of the blot.
(TIF)

Figure S3 ***grau;mtrm/+*** **spindles are meiotic in structure.** (A) An egg laid by a *grau^QQ36/RM61^;mtrm^126^/+* female is shown. A free centrosome (presumably deposited by the sperm) is indicated by the arrow. Although the free centrosome shows the presence of both alpha-and gamma-tubulin, the spindles contained in the egg are not enriched for gamma-tubulin at their poles. Scale bar represents 50 um. (B) Mitotically dividing embryo from an *OrR* female. Centrosomes are readily detected by the presence of gamma-tubulin at the spindle poles. Scale bar represents 50 um.
(TIF)

Figure S4 **Cort is not restored in** ***grau^QQ36/RM61^; mtrm^126^/+ mutants*** **.** The partial suppression of the *grau* phenotype in *grau^QQ36/RM61^; mtrm^126^/+* activated eggs is not due to restoration of Cort protein. Western blot showing presence of Cort in *grau/CyO* ovaries but not *grau* or *grau; mtrm^126^/+* ovaries. Cortex levels are also not restored in *grau^QQ36/RM61^; mtrm^126^/+* fertilized eggs. The asterisk indicates a nonspecific band. Ovary and fertilized egg panels are from two separate blots. Molecular weight markers are indicated to the side of the blot.
(TIF)

Figure S5 **Comparison of Mtrm protein levels from various transgenic lines.** (A) Western blot showing protein amounts from the indicated genotypes. (UAS) 3×FLAG-Mtrm is seen at higher levels than stabilized mCherry-Mtrm (expressed from the endogenous *mtrm* promoter) in both stage 14 oocytes and activated, fertilized eggs (collected for 1 h and left to develop for 1 h in A). Molecular weight markers are indicated to the side of the blot. (B) Activated eggs were collected for 30 min and left to develop for 2 or 3 h. Molecular weight markers are indicated to the side of the blot. Stg. 14 s and activated eggs are from two different blots. (C) Activated eggs were collected/aged as indicated. Molecular weight markers are indicated at the side of the blot. Stg. 14 s and activated eggs are from two different blots.
(TIF)

Table S1 **Immunoprecipitation of Cortex identifies APC/C components and Matrimony.** Data summarizing three independent IP/mass spec experiments are shown. The number of total spectra identified that immunoprecipitated/Co-IP'd with Cortex is indicated. The number of peptides identified in the negative control is shown in parentheses. In experiments 1 and 2, random mouse IgG was used as a negative control. Experiment 3 used anti-myc antibody in a strain not expressing 6×Myc-Cortex (*OrR*) as a control. *: Number of spectra indicated were searched for in MASCOT and analyzed by Scaffold (see Materials and Methods for more details).
(DOCX)

Table S2 **Quantification of mitotic index in the Cortex stable line.** Cells were induced (or not) with 0.5 mM CuSO4. Total cells were counted using DAPI, and mitotic cells were counted using anti-phospho histone H3.
(DOCX)

Table S3 **Analysis of Cortex stable line by FACS.** The stable Cortex cell line or Kc167 cells were incubated with or without CuSO_4_ and cell cycle progression was analyzed by FACs (after 1 or 2 d of treatment). Cells are predominantly in G2, as is typical of Kc cells [56]. No significant cell cycle arrest is induced by ectopic expression of Cortex. A significant arrest in G2 was detected when MG132 was added to the medium for 8 h.
(DOCX)

Table S4 **Mtrm-4A and L21A are competent to rescue chromosome nondisjunction in** ***mtrm/+*** **heterozygotes.** Both mCherry-Mtrm-L21A and 4A can rescue nondisjunction caused by heterozygous deletion of *mtrm*. *: Full genetic background is *FM7w/yw; transgene/+; nanos-GAL4:VP16, mtrm^Df(3L)66C-T2-10^/+; spa^pol^*. **Adjusted totals were calculated as in Hawley et al. [57].
(DOCX)

## Abbreviations

APC/C: Anaphase Promoting Complex/Cyclosome
