# Supplementary figures and images for: A Meiosis-Specific Form of the APC/C Promotes the Oocyte-to-Embryo Transition by Decreasing Levels of the Polo Kinase Inhibitor Matrimony
Source: PLoS Biol. 2013 Sep 3;11(9):e1001648. doi: 10.1371/journal.pbio.1001648 (PMC3760765; doi:10.1371/journal.pbio.1001648)

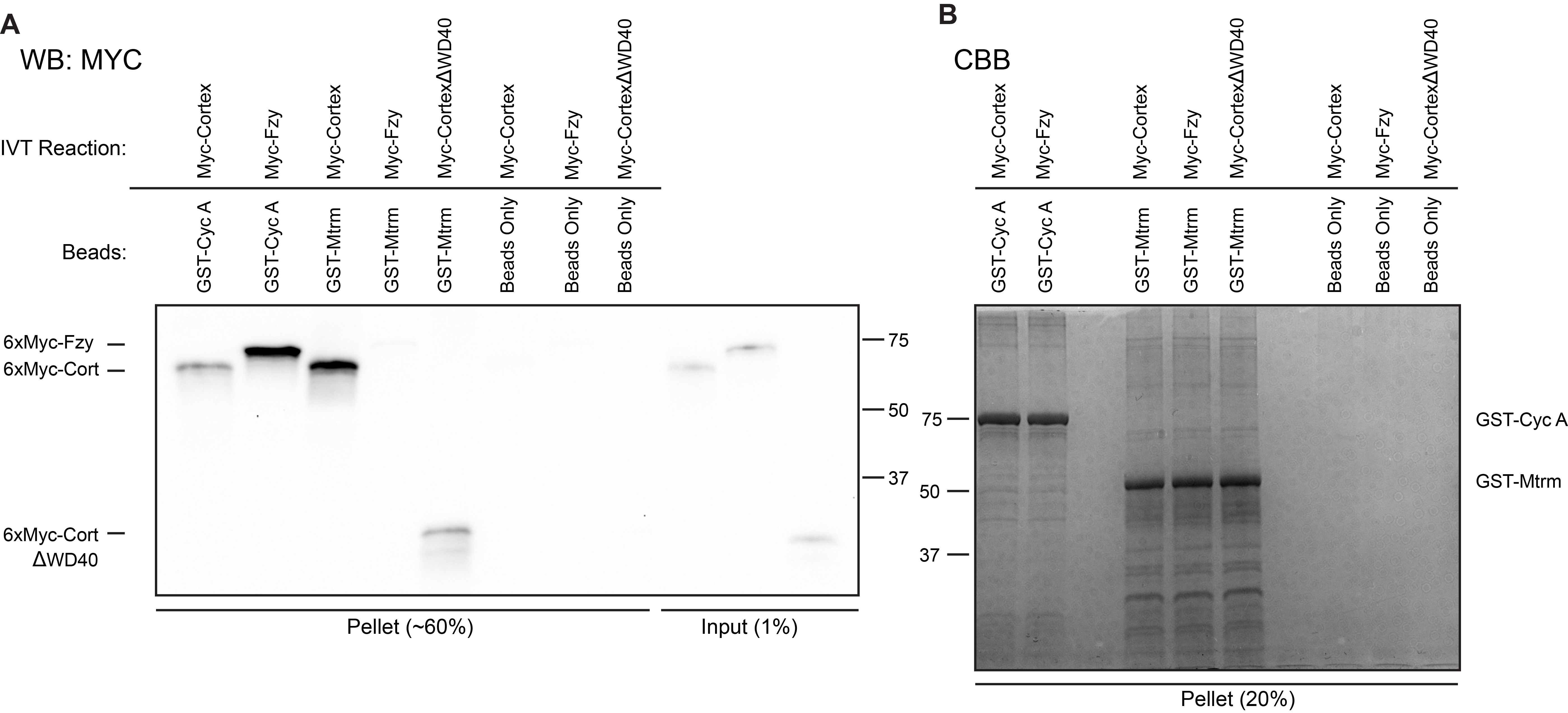

Supplement: Figure S1 — In vitro binding assays with Cyclin A and CortΔWD40. (A) Western blot showing in vitro translated Myc-tagged Fzy/Cdc20 stably binds to GST-CycA. Myc-Cortex also binds, but somewhat less efficiently. Myc-CortexΔWD40 (AA 1–148 of Cortex) is impaired in its ability to bind GST-Mtrm. Glutathione beads alone serve as a negative control. Quantification indicates Myc-Fzy binds to GST-CycA 155× better than to GST-Mtrm. 6×Myc-Cortex (full length) binds GST-Mtrm 5.8× better than 6×Myc-CorttΔWD40. About 60% of each pellet sample was subjected to SDS-PAGE followed by Western blotting (remaining pellet sample was used for B). Right side of panel shows 1% of total input of in vitro translated 6×MycCort, 6×MycFzy/Cdc20, and 6×Myc-CortΔWD40. Blot was probed with anti-Myc (9E10) antibody. Molecular weight markers are indicated to the side of the blot. (B) Coomassie stain of purified proteins used in binding assay. 20% of the final washed pellet was subjected to SDS-PAGE followed by Coomassie staining. Molecular weight markers are indicated to the side of the gel. (TIF) [file pbio.1001648.s001.tif]

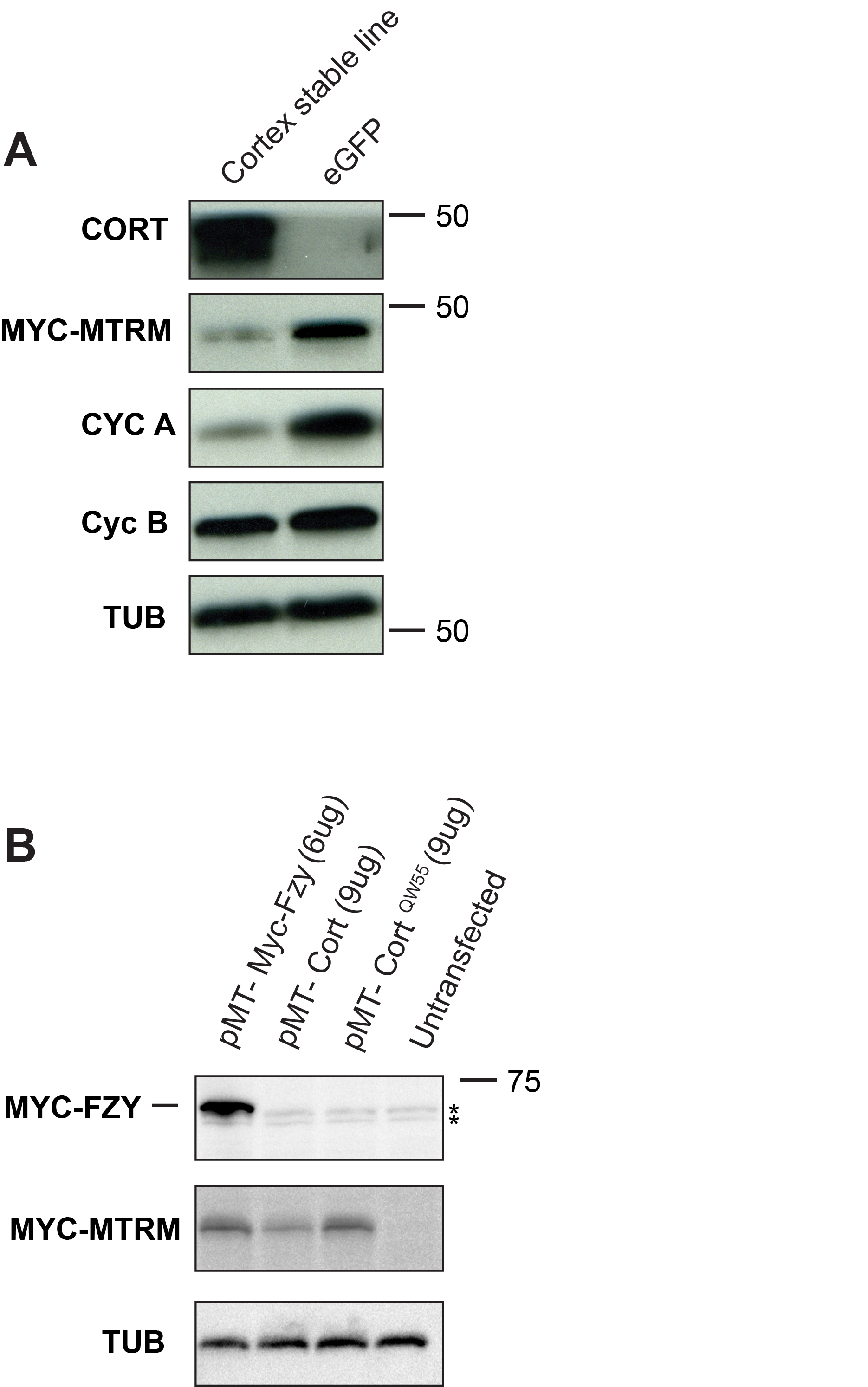

Supplement: Figure S2 — Levels of cell cycle proteins in cell culture system. (A) A cell line with a stable cort gene shows decreased Cyclin protein levels. Western blots comparing levels of indicated proteins in a cort stable line and cells transfected with pMT-eGFP instead. Both populations were also transfected with pMT-6×myc-mtrm. Molecular weight markers are indicated to the side of the blot. (B) Expression of myc-tagged Fizzy/Cdc20 does not decrease myc-tagged Mtrm levels. Amount of plasmid used to transfect cells is indicated above each lane. Cells were also transfected with equal amounts of pMT-6×myc-mtrm (except last lane). The asterisks indicate nonspecific bands. Both Myc-Fzy and Myc-Mtrm were detected using anti-myc antibodies. Molecular weight markers are indicated to the side of the blot. (TIF) [file pbio.1001648.s002.tif]

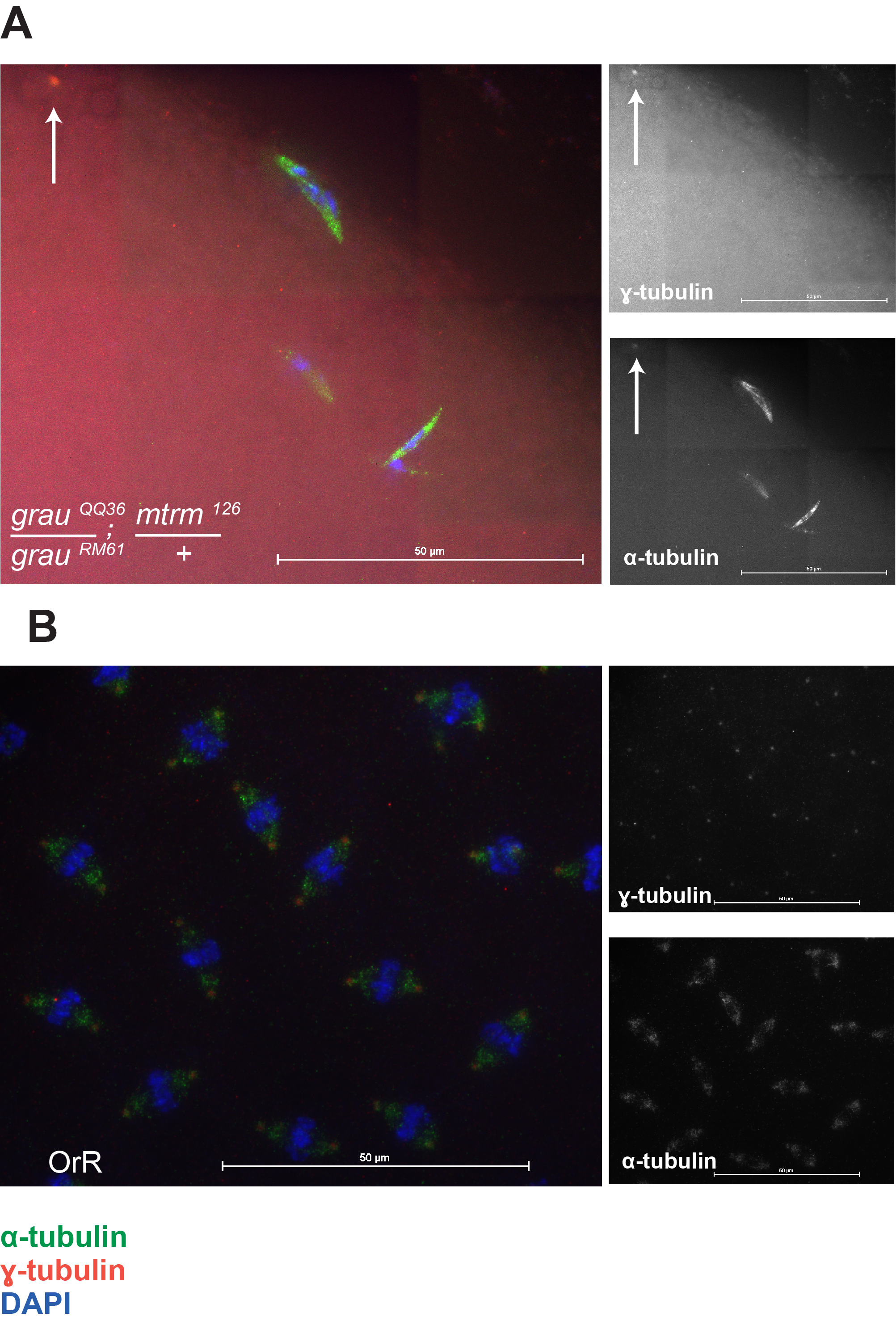

Supplement: Figure S3 — grau;mtrm/+ spindles are meiotic in structure. (A) An egg laid by a grauQQ36/RM61;mtrm126/+ female is shown. A free centrosome (presumably deposited by the sperm) is indicated by the arrow. Although the free centrosome shows the presence of both alpha-and gamma-tubulin, the spindles contained in the egg are not enriched for gamma-tubulin at their poles. Scale bar represents 50 um. (B) Mitotically dividing embryo from an OrR female. Centrosomes are readily detected by the presence of gamma-tubulin at the spindle poles. Scale bar represents 50 um. (TIF) [file pbio.1001648.s003.tif]

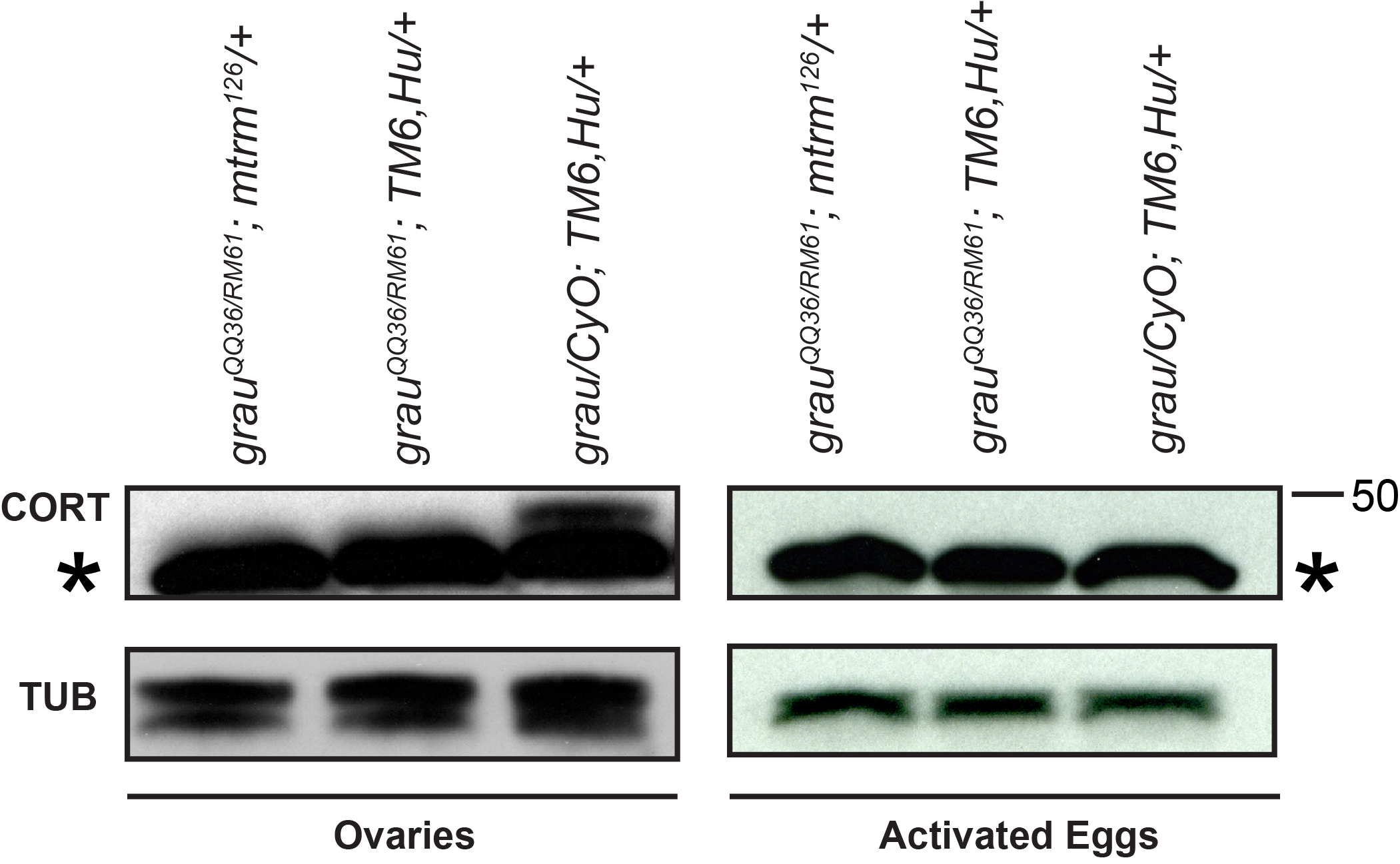

Supplement: Figure S4 — Cort is not restored in grauQQ36/RM61; mtrm126/+ mutants . The partial suppression of the grau phenotype in grauQQ36/RM61; mtrm126/+ activated eggs is not due to restoration of Cort protein. Western blot showing presence of Cort in grau/CyO ovaries but not grau or grau; mtrm126/+ ovaries. Cortex levels are also not restored in grauQQ36/RM61; mtrm126/+ fertilized eggs. The asterisk indicates a nonspecific band. Ovary and fertilized egg panels are from two separate blots. Molecular weight markers are indicated to the side of the blot. (TIF) [file pbio.1001648.s004.tif]

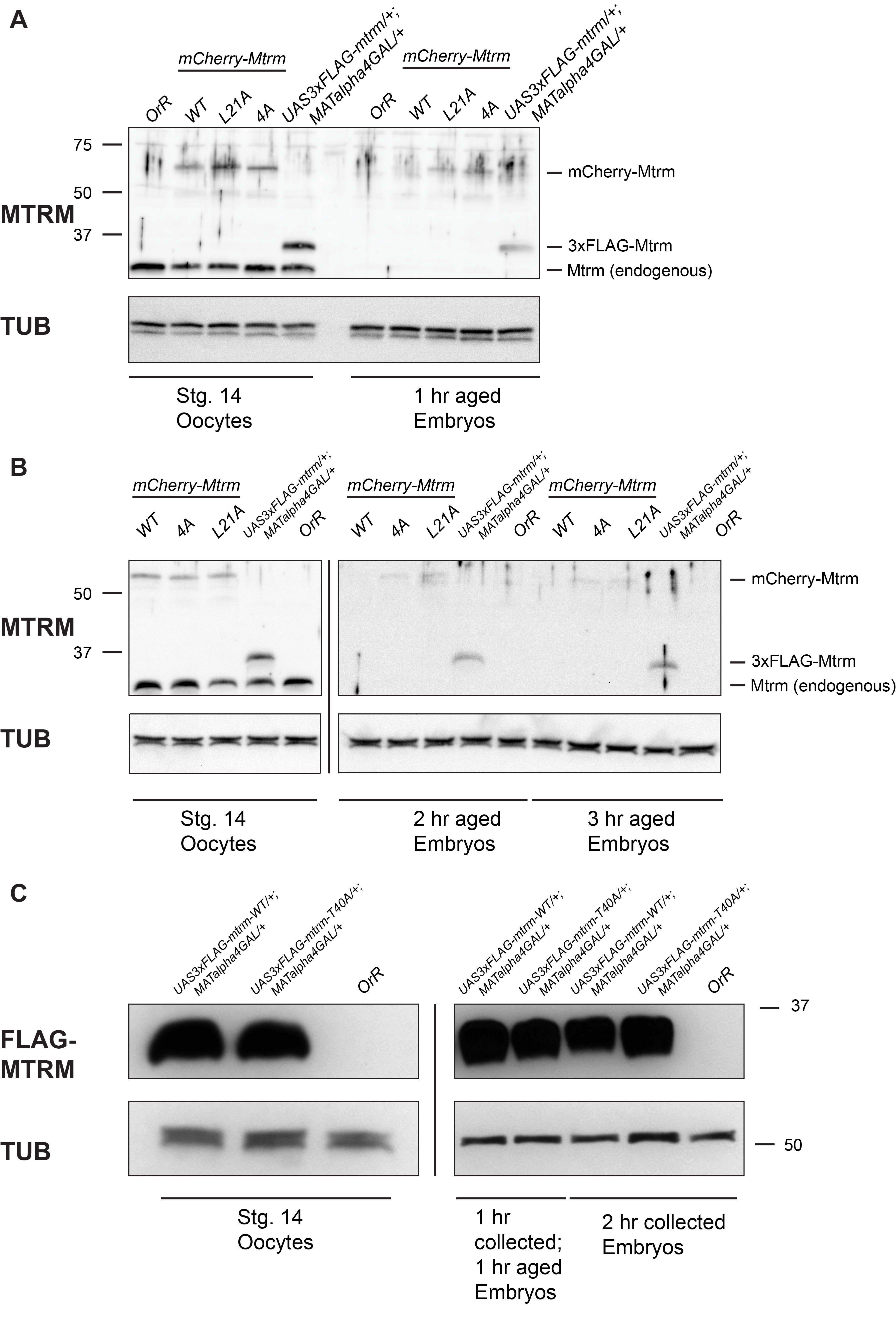

Supplement: Figure S5 — Comparison of Mtrm protein levels from various transgenic lines. (A) Western blot showing protein amounts from the indicated genotypes. (UAS) 3×FLAG-Mtrm is seen at higher levels than stabilized mCherry-Mtrm (expressed from the endogenous mtrm promoter) in both stage 14 oocytes and activated, fertilized eggs (collected for 1 h and left to develop for 1 h in A). Molecular weight markers are indicated to the side of the blot. (B) Activated eggs were collected for 30 min and left to develop for 2 or 3 h. Molecular weight markers are indicated to the side of the blot. Stg. 14 s and activated eggs are from two different blots. (C) Activated eggs were collected/aged as indicated. Molecular weight markers are indicated at the side of the blot. Stg. 14 s and activated eggs are from two different blots. (TIF) [file pbio.1001648.s005.tif]
