# Supplementary material for: A Meiosis-Specific Form of the APC/C Promotes the Oocyte-to-Embryo Transition by Decreasing Levels of the Polo Kinase Inhibitor Matrimony
Source: PLoS Biol. 2013 Sep 3;11(9):e1001648. doi: 10.1371/journal.pbio.1001648 (PMC3760765; doi:10.1371/journal.pbio.1001648)
Supplement: Table S1 — Immunoprecipitation of Cortex identifies APC/C components and Matrimony. Data summarizing three independent IP/mass spec experiments are shown. The number of total spectra identified that immunoprecipitated/Co-IP'd with Cortex is indicated. The number of peptides identified in the negative control is shown in parentheses. In experiments 1 and 2, random mouse IgG was used as a negative control. Experiment 3 used anti-myc antibody in a strain not expressing 6×Myc-Cortex (OrR) as a control. *: Number of spectra indicated were searched for in MASCOT and analyzed by Scaffold (see Materials and Methods for more details). (DOCX) [file pbio.1001648.s006.docx]

|  | Experiment 1* | Experiment 2* | Experiment 3* |
| --- | --- | --- | --- |
| Cortex | 98(0) | 118(2) | 102(0) |
| Cdc16 | 9(0) | 8(0) | 4(0) |
| Cdc27 | 0(0) | 4(0) | 5(0) |
| Cdc23 | 3 (0) | 4 (0) | 5 (0) |
| Shattered/Apc1 | 10(0) | 7(0) | 5(0) |
| Matrimony | 2 (0) | 2(0) | 0 (0) |
