# Supplementary material for: A Meiosis-Specific Form of the APC/C Promotes the Oocyte-to-Embryo Transition by Decreasing Levels of the Polo Kinase Inhibitor Matrimony
Source: PLoS Biol. 2013 Sep 3;11(9):e1001648. doi: 10.1371/journal.pbio.1001648 (PMC3760765; doi:10.1371/journal.pbio.1001648)
Supplement: Table S2 — Quantification of mitotic index in the Cortex stable line. Cells were induced (or not) with 0.5 mM CuSO4. Total cells were counted using DAPI, and mitotic cells were counted using anti-phospho histone H3. (DOCX) [file pbio.1001648.s007.docx]

|  | Total # Cells | # Cells PH3 + | % PH3 + |
| --- | --- | --- | --- |
| Cortex Stable Line (Induced) | 811 | 24 | 2.96 |
| Cortex Stable Line (Uninduced) | 1452 | 45 | 3.10 |
