# Supplementary material for: A Meiosis-Specific Form of the APC/C Promotes the Oocyte-to-Embryo Transition by Decreasing Levels of the Polo Kinase Inhibitor Matrimony
Source: PLoS Biol. 2013 Sep 3;11(9):e1001648. doi: 10.1371/journal.pbio.1001648 (PMC3760765; doi:10.1371/journal.pbio.1001648)
Supplement: Table S3 — Analysis of Cortex stable line by FACS. The stable Cortex cell line or Kc167 cells were incubated with or without CuSO4 and cell cycle progression was analyzed by FACs (after 1 or 2 d of treatment). Cells are predominantly in G2, as is typical of Kc cells [56]. No significant cell cycle arrest is induced by ectopic expression of Cortex. A significant arrest in G2 was detected when MG132 was added to the medium for 8 h. (DOCX) [file pbio.1001648.s008.docx]

| Cell Type | CuSO4? | %G1 | %S | %G2 |
| --- | --- | --- | --- | --- |
| Cortex Stable Line | 0.5 mM CuSO4 (1 day) | 13.3 | 6.15 | 67.4 |
| Kc167 | 0.5 mM CuSO4 (1 day) | 11 | 7.18 | 67.7 |
| Cortex Stable Line | No CuSO4 (1 day) | 15.7 | 11.9 | 56.4 |
| Kc167 | No CuSO4 (1 day) | 17.8 | 13.7 | 51.7 |
| Cortex Stable Line | 0.5 mM CuSO4 (2 day) | 16.6 | 9.12 | 61.7 |
| Kc167 | 0.5 mM CuSO4 (2 day) | 14.1 | 9.7 | 62.7 |
| Kc167 | 0.5 mM CuSO4 (1 day) + MG132 (25uM; 8hrs) | 3.78 | 0 | 88.7 |
