# Supplementary material for: A Meiosis-Specific Form of the APC/C Promotes the Oocyte-to-Embryo Transition by Decreasing Levels of the Polo Kinase Inhibitor Matrimony
Source: PLoS Biol. 2013 Sep 3;11(9):e1001648. doi: 10.1371/journal.pbio.1001648 (PMC3760765; doi:10.1371/journal.pbio.1001648)
Supplement: Table S4 — Mtrm-4A and L21A are competent to rescue chromosome nondisjunction in mtrm/+ heterozygotes. Both mCherry-Mtrm-L21A and 4A can rescue nondisjunction caused by heterozygous deletion of mtrm. *: Full genetic background is FM7w/yw; transgene/+; nanos-GAL4:VP16, mtrmDf(3L)66C-T2-10/+; spapol. **Adjusted totals were calculated as in Hawley et al. [57]. (DOCX) [file pbio.1001648.s009.docx]

| Genotype | Adjusted Total** | %*X* NDJ | % *4th* NDJ |
| --- | --- | --- | --- |
| *FM7w/yw; spa^pol^* | 1117 | 2.1 | 0.6 |
| No rescue construct^*^ | 1572 | 35.8 | 20.2 |
| *mtrm^WT*^* | 1574 | 1.4 | 2.6 |
| *mtrm^L21A*^* | 2193 | 1.1 | 2.6 |
| *mtrm^4A*^* | 1540 | 1 | 3.9 |
